# CD163 Expression Was Associated with Angiogenesis and Shortened Survival in Patients with Uniformly Treated Classical Hodgkin Lymphoma

**DOI:** 10.1371/journal.pone.0087066

**Published:** 2014-01-29

**Authors:** Young Wha Koh, Chan-Sik Park, Dok Hyun Yoon, Cheolwon Suh, Jooryung Huh

**Affiliations:** 1 Department of Pathology, Ulsan University Hospital, University of Ulsan College of Medicine, Ulsan, South Korea; 2 Department of Pathology, Asan Medical Center, University of Ulsan College of Medicine, Seoul, South Korea; 3 Department of Oncology, Asan Medical Center, University of Ulsan College of Medicine, Seoul, South Korea; University of Modena & Reggio Emilia, Italy

## Abstract

**Background:**

Recent studies have reported the prognostic value of tissue-associated magrophages (TAMs) in classical Hodgkin lymphoma (cHL). In addition, TAMs are implicated in the tumor angiogenesis. In this study, we examined the prognostic relevance of TAMs in relation to vascular endothelial growth factor (VEGF) expression and angiogenesis in uniformly treated cases of cHL.

**Methods:**

Diagnostic tissue from 116 patients with ABVD-treated cHL was evaluated retrospectively by immunohistochemical analysis for CD68, CD163 and VEGF expression and for CD31 expression as a measure of microvessel density (MVD).

**Results:**

High CD163 expression (≥35% of cellularity) correlated with VEGF expression (Pearson’s Chi-square test, *P* = 0.008) and MVD (Spearman correlation coefficient 0.310, *P*<0.001). High CD163 expression was associated with inferior event-free survival (EFS, *P* = 0.005) and overall survival (OS, *P*<0.001) in univariate analysis. In multivariate analysis, high CD163 expression was strongly associated with inferior EFS (*P* = 0.043) and OS (*P* = 0.008). Patients with high MVD had a lower OS than those with low MVD, but the difference was not significant *(P* = 0.071, respectively). While high expression of CD68 was also associated with inferior EFS (*P* = 0.007), it showed no correlation with VEGF or MVD.

**Conclusions:**

Our data confirms that CD163 expression provides independent prognostic information in cHL. The correlation of CD163 with VEGF expression and MVD suggests the role of CD163-positive cells in tumor angiogenesis of cHL.

## Introduction

Classical Hodgkin lymphoma (cHL) is characterized by the disruption of the normal lymph node architecture by the presence of few of Hodgkin/Reed-Sternberg (HRS) cells in a background of reactive bystander cells mainly composed of T and B lymphocytes, macrophages and other cell types [1]. cHL is associated with high cure rates; However, despite significant advances in treatment, there remains a significant minority of patients with refractory disease in whom prolonged exposure to initial therapy induces chemo-resistance and unnecessary toxicity [2]. The major challenge remains to tailor treatments to eradicate cHL with minimal side-effects and to find biological predictive markers for patients who need intensive therapy [2].

The tumor microenvironment is emerging as an important player in the progression of malignant tumors including cHL. Recently, tumor-associated macrophages (TAMs) in lesional tissues have been shown to be a strong prognostic indicator of cHL by gene expression profile analysis and subsequent immunohistochemical detection using CD68 and CD163 as markers [3]–[6]. Furthermore, the peripheral blood lymphocyte/monocyte ratio at diagnosis in cHL was reported to be a prognostic factor of clinical outcome [7], [8].

Vascular endothelial growth factor (VEGF) plays an important role in physiologic and pathologic angiogenesis, including neoangiogenesis in tumors [9], [10]. VEGF expression has also demonstrated prognostic value in several solid malignancies [11], [12]. Bevacizumab is a humanized VEGF antagonist approved by the Food and Drug Administration for use in the treatment of human solid cancers [13], [14]. By blocking VEGF binding to the VEGF receptor, bevacizumab interferes with tumor angiogenesis. VEGF inhibition has shown significant survival benefit in several types of solid malignancies. Previous studies have established that VEGF is expressed in cHL [15], [16].

CD31 is a reliable marker of the vascular endothelium. Quantification of CD31 stained vessels in tumors is a standard method of measuring of intra-tumoral microvessel density (MVD) [17], a useful prognostic indicator in various malignant tumors. [18], [19] MVD is correlated with the biologic behavior of non-Hodgkin’s lymphoma [20]–[22] as well as cHL [16], [23], [24].

Although the mechanisms by which TAMs affect cancer progression are still unclear and are probably multifactorial, one of the potential tumor-promoting functions of TAMs is as a proangiogenetic factor via the expression of angiogenic factors, such as VEGF [25], [26]. A recent study reported a positive correlation between CD68-positive TAMs and MVD in cHL [27]. However, CD163 may be a superior marker of TAMs due to its higher specificity, as compared to CD68, for M2 macrophages [28], which are involved in tumor angiogenesis and progression, [29], [30]. While associations between TAMs, VEGF and MVD have been observed in several malignancies [31]–[34], no study has examined the relationship and prognostic implication of CD68, CD163, and VEGF expression and MVD in cHL patients. This retrospective study evaluated CD68, CD163, and VEGF expression and MVD in cHL patients to determine correlations between these markers and assess their prognostic significance.

## Materials and Methods

### Patients

The present research was approved by the Internal Review Board of the Asan Medical Center. No informed consent (written or verbal) was obtained for use of retrospective tissue samples from the patients within present study, the IRB waived the need for written informed consent. This retrospective study reviewed histological and immunohistochemical data from 116 consecutive patients diagnosed with cHL at Asan Medical Center, Seoul, South Korea, between 1990 and 2012. All patients were ≥15 years of age at diagnosis, had pathologically confirmed cHL, no previous treatment, no history of malignancy and had been treated with doxorubicin, bleomycin, vinblastine, and dacarbazine (ABVD) therapy regimens, with or without radiation. Paraffin-embedded tumor tissues and follow-up data were available for all included patients.

The median follow-up time was 6.167 years (interquartile range: 3.75–10.33 years). Response criteria were based on standard guidelines. Routine follow-up imaging analyses were performed every 3 months for the first 2 years, every 6 months for the next 3 years, and then annually (or whenever clinically indicated) thereafter.

### Histopathological Analysis and Immunohistochemistry

All histological and immunophenotypic data of the 116 patients with cHL were reviewed by two pathologists (JH and YWK). According to the World Health Organization (WHO) criteria, the cases were subtyped as follows: nodular sclerosis (NS), lymphocyte-rich (LR), mixed cellularity (MC), lymphocyte-depleted (LD), or not otherwise specified cHL. A tissue microarray (TMA) was generated with three 1 mm diameter tumor cores from selected areas of each formalin-fixed paraffin-embedded tumor sample. TMA section were stained using an automatic immunohistochemistry staining device (Benchmark XT, Ventana Medical System, Tucson, AZ, USA). Briefly, 5 µm thick sections were transferred onto poly-L-lysine-coated adhesive slides and dried at 62°C for 30 minutes. After standard heat epitope retrieval for 30 minutes in ethylenediaminetetraacetic acid (pH 8.0), the samples were incubated with antibodies against cleaved CD68 (dilution 1∶2000, DAKO, Glostrup, Denmark), CD163 (dilution 1∶400, NOVO, Newcastle, UK), VEGF (dilution 1∶500; Pharmingen, New Jersey, USA), CD31 (dilution 1∶100; Novo, Newcastle, UK) and CD30 (1∶25 dilution, clone BER-H2, mouse monoclonal; DAKO). The sections were then incubated with biotinylated anti-mouse immunoglobulins, peroxidase-conjugated streptavidin (LSAB kit, DAKO, Glostrup, Denmark), and 3,3′-diaminobenzidine. Slides were counterstained with Harris hematoxylin.

Each case was represented by three tissue cores in the TMA, with at least ten HRS cells detected in at least one of the three core cylinders from each patient. To minimize the counting of non-specific staining in cells other than macrophages, we only counted staining in cells that were morphologically compatible with macrophages, avoiding fibroblast, endothelial cell and Hodgkin-Reed-Sternberg cells on the basis of their size, shape, and CD30 staining. We examined protein expression levels of CD68, CD163 and VEGF in 5% increment. The relative percentage of CD68-positive or CD163-positive cells relative to overall cellularity was reported as mean scores. In cases with >10% difference in scores awarded by the two pathologists, re-evaluation was performed using a double-headed microscope. The pathologists agreed on the level of CD68 expression (<30% vs. ≥30%) in 99 out of 116 cases (85.3%, k = 0.644), on the level of CD163 expression (<35% vs. ≥35%) in 102 out of 116 cases (87.9%, k = 0.662). Cutoff values for CD68, CD163, and VEGF expression that showed the most significant differences in OS were selected (Table S1). A sample was considered high-CD68 expression if positive cells made up 30% or more of the overall cellularity and high-CD163 expression if positive cells made up 35% or more of the overall cellularity. A sample was considered VEGF-positive if 25% or more of the HRS cells showed reactivity to the VEGF antibody, while a sample was considered VEGF-negative if VEGF expression was detected in <25% of the HRS cells, or in bystander cells only. CD30 stain was used as a guide to identify HRS cells for the interpretation of VEGF.

To quantify MVD, the areas of highest vascularization were selected at low power (×100), and quantified at high magnification (×400). Three hot spots were selected per case and quantified simultaneously by two pathologists. The final MVD for each case was the mean of the number of vessels counted in the three hot spots that were scored. Microvessels with a clearly defined lumen or with a well-defined linear vessel shape were included in the counts. For statistical analysis, the cases were divided into low and high MVD groups, with high MVD tumors having ≥15.33 vessels/field according to the maximal chi-square method.

*In situ* hybridization (ISH) analysis for EBV-encoded RNA-1 and RNA-2 (EBER) was performed and scored as described elsewhere [35].

### Statistical Analysis

OS was defined as the interval between the date of diagnosis and the date of death from any cause. Follow-up of living patients (with or without events) was censored at their last follow-up date. Event-free survival (EFS) was defined as the interval between the date of diagnosis and the date of disease progression, relapse, or death from any cause. Cumulative OS and EFS were analyzed by the Kaplan-Meier method, with comparisons analyzed by log-rank testing.

Multivariate prognostic analyses were performed on OS and EFS with the Cox proportional hazards regression model using the enter method. Categorical variables were compared using the chi-square test. Continuous variables were compared using the Mann–Whitney U test and Spearman’s correlation coefficients were used to evaluate associations for continuous variables. The maximal chi-square method was used to determine the cutoff of MVD. The maximal chi-square method was adopted to evaluate which cutoff point in each data set best segregated patients into poor and good prognosis subgroups (based on the likelihood of survival), with the log-rank test as the method used to measure the strength of the grouping [36], [37]. All statistical analyses were performed using the SPSS statistical software program (version 18.0; SPSS, Chicago, IL) or R 2.15.2. All P values are two-sided associations and P<0.05 is considered statistically significant.

## Results

### Patient Characteristics

The clinical characteristics of the 116 patients included in the study are summarized in Table 1. Patient age ranged from 15 to 77 years (median: 35 years). Forty-four patients experienced relapse, disease progression, or death, and 20 patients died. Median OS and EFS were not reached. The estimated 5-year OS and EFS were 83.7% and 58.9%, respectively.

**Table 1:** Demographic and clinical characteristics of patients.

Characteristics at diagnosis	No. of patients (%)
Age, median (range, years)	35 (15–77)
Male gender	68 (58.6%)
Histologic subtype	
Nodular sclerosis	78 (67.2%)
Mixed cellularity	22 (19%)
Lymphocyte-rich	5 (4.3%)
Lymphocyte-depleted	3 (2.6%)
Not classifiable	8 (6.9%)
Ann Arbor stage	
I	21 (18.1%)
II	41 (35.3%)
III	27 (23.3%)
IV	27 (23.3%)
Stage (limited vs. advanced)	
Limited	45 (38.8%)
Advanced	71 (61.2%)
B symptoms present	37 (31.9%)
International Prognostic Score ≥3 (high-risk)	45 (38.8%)
EBER positivity	43 (37.1%)
Primary treatment	
Chemotherapy	85 (73.3%)
Chemoradiotherapy	31 (26.7%)

EBER, Epstein-Barr virus-encoded RNA-1 and RNA-2 assessed by *in situ* hybridization.

### CD68, CD163, VEGF, and CD31 Expression in cHL Tissues

Correlations of CD68, CD163, VEGF, and MVD with clinical variables are summarized in Table S2.

**Figure 1:**
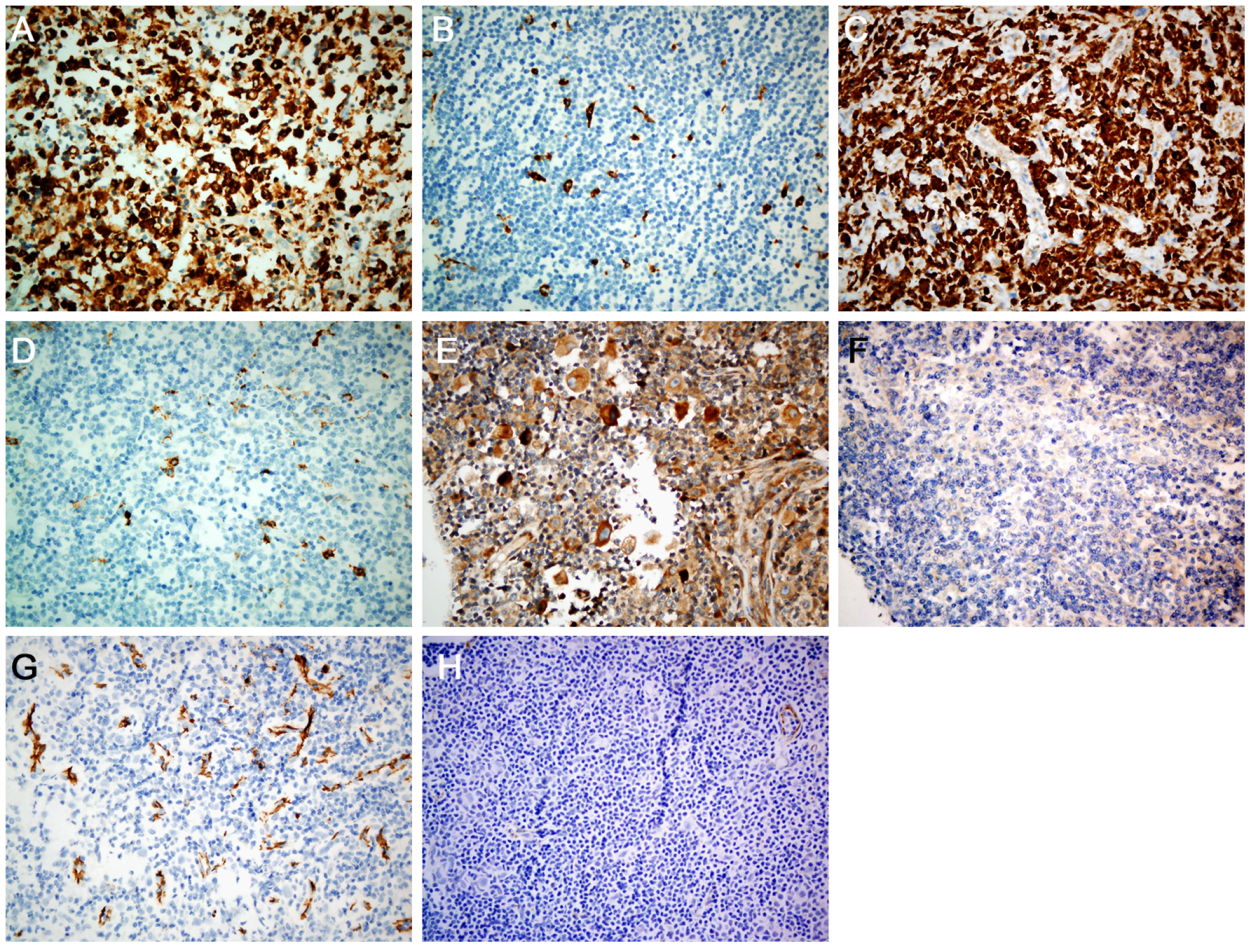
CD68, CD163, vascular endothelial growth factor (VEGF), and CD31 expression in cHL tissues. (A) High CD68 expression (≥30%). (B) Low CD68 expression (<30%). (C) High CD163 expression (≥35%). (D) Low CD163 expression (<35%). (E) High VEGF expression (≥25%). (F) Low VEGF expression (<25%). (G) High microvessel density with CD31 expression. (H) Low microvessel density with CD31 expression.

The high-CD68 expression group (CD68≥30%, n = 32, Fig. 1A) included more men (78.1% vs. 51.2%, *P = *0.011), high risk IPS patients (56.3% vs. 32.1%, *P* = 0.02), and cases of EBER positivity (53.1% vs. 31%, *P* = 0.033) compared to the low-CD68 group (CD68<30%, n = 84, Fig. 1B).

The high-CD163 group (CD163≥35%, n = 26, Fig. 1C) included more patients who were older (69.2% vs. 32.2%, *P*<0.001), of the male gender (84.6% vs. 51.1%, *P* = 0.003), had high risk IPS (69.2% vs. 30%, *P*<0.001), and who showed EBER positivity (61.5% vs. 30%, *P* = 0.005) compared to the low-CD163 group (CD163<35%, n = 90, Fig. 1D). A statistically significant correlation was observed between high CD68 and CD163 expression (*P*<0.001).

Neither the high-VEGF expression (VEGF ≥25%, n = 33, Fig. 1E) nor the low-VEGF expression (VEGF <25%, n = 83, Fig. 1F) groups were associated with clinical variables.

The mean MVD of all of cases was 13 (standard deviation [SD] = 6.82), with a range of 1–40. Forty-three samples had high MVD (MVD≥15.33, Fig. 1G), while the remaining 73 had low MVD (MVD<15.33, Fig. 1H). Low MVD was significantly associated with high levels of LDH (65.8% vs. 44.2%, *P = *0.032).

### Correlations of CD68, CD163 and VEGF Expression with MVD

A statistically significant correlation was observed between high CD163 and VEGF expression (*P* = 0.008, Table 2), and between high MVD and VEGF expression (*P* = 0.019, Table 2). There was no correlation between CD68 index and VEGF expression (*P* = 0.106).

**Table 2:** Correlations among CD68 expression, CD163 expression, VEGF expression, and microvessel density.

CD68 expression	VEGF expression		*P*-value
	Negative (n = 83)	Positive (n = 33)	0.106†
Low (n = 84)	64 (77.1%)	20 (60.6%)	
High (n = 32)	19 (22.9%)	13 (39.4%)	
**CD163 expression**	**VEGF expression**		
	**Negative (n = 83)**	**Positive (n = 33)**	**0.008** †
Low (n = 90)	70 (84.3%)	20 (60.6%)	
High (n = 26)	13 (15.7%)	13 (39.4%)	
**MVD**	**VEGF expression**		
	**Negative (n = 83)**	**Positive (n = 33)**	**0.019** †
Low (n = 73)	58 (69.9%)	15 (45.5%)	
High (n = 43)	25 (30.1%)	18 (54.5%)	

VEGF, vascular endothelial growth factor; MVD, microvessel density.

† Chi-squared test by two-sided Pearson’s test.

We performed a correlation study on the relationship between CD68 expression, CD163 expression and MVD. There was a positive correlation between CD163 index and MVD in cHL tissues as assessed by Spearman correlation analysis regression (rho = 0.310, *P*<0.001, Fig. S1A). No correlation between MVD and CD68 expression was identified by Spearman correlation analysis regression (*P* = 0.176, Fig. S1B).

### Prognostic Significance of CD68, CD163, and VEGF Expression and MVD

**Figure 2:**
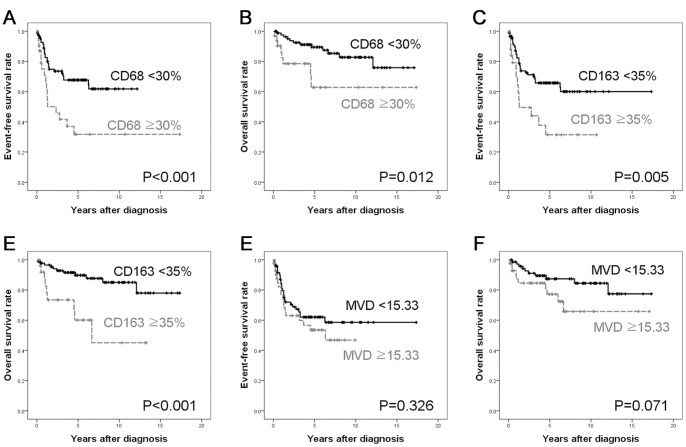
Comparison of survival rates according to CD68, CD163, VEGF expression and MVD. Event-free survival (EFS) (A) or overall survival (OS) (B) was significantly worse in high-CD68 group. EFS (C) or OS (D) was significantly worse in high-CD163 group. MVD was not significantly associated with EFS (E). Patients with high MVD had worse OS (F) than those with low MVD, although the statistical significance was not reached.

High-CD68 groups had lower 5-year EFS (31.7% vs. 67.7%, *P*<0.001; Fig. 2A) and 5-year OS (62.8% vs. 89.4%, *P* = 0.012; Fig. 2B) rates than low-CD68 patients. The high-CD163 group had lower 5-year EFS (31.4% vs. 65.7%, *P* = 0.005; Fig. 2C) and 5-year OS (60.1% vs. 89.8%, *P*<0.001; Fig. 2D) rates than low-CD163 patients. VEGF expression was not significantly associated with either EFS or OS (*P* = 0.342 and *P* = 0.339, respectively). Patients with high MVD had worse OS than those with low MVD (5-year OS, 77.2% vs. 87.4%; *P* = 0.071, Fig. 2F), although statistical significance was not reached. MVD was not significantly associated with EFS (*P* = 0.326, Fig. 2E).

Patients with high risk IPS (≥3) had lower 5-year OS rates compared to patients with low risk (71.6% vs. 91.2%, *P* = 0.01; Fig. S2A), however high risk IPS (≥3) was not associated with EFS rates (*P* = 0.098; Fig. S2B).

By univariate analysis, both OS and EFS were associated with IPS (≥3). CD68 and CD163 indices were associated with EFS and OS (Table 3). By multivariate analysis, CD68 and CD163 expression were independent prognostic markers for EFS (*P* = 0.007 and *P* = 0.034, respectively, Table 4). High CD163 expression was independent prognostic marker for OS (*P* = 0.026 Table 4), along with high risk IPS (≥3).

**Table 3:** Univariate analysis for overall survival (OS) and event-free survival (EFS).

		OS			EFS		
Covariate	Subcategory	HR	95% CI	*P*-value	HR	95% CI	*P*-value
B symptoms	(−) vs. (+)	1.795	0.74–4.35	0.196	1.292	0.69–2.38	0.414
IPS	<3 vs. ≥3	3.314	1.24–7.86	0.015	1.635	0.90–2.95	0.104
LDH (U/L)	Normal vs. abnormal	1.430	0.50–4.03	0.499	0.981	0.57–1.96	0.855
EBER	(−) vs. (+)	1.624	0.66–3.93	0.284	1.634	0.90–2.95	0.105
CD68 expression	(−) vs. (+)	3.071	1.22–7.67	0.016	2.583	1.40–4.76	0.002
CD163 expression	(−) vs. (+)	4.148	1.70–10.1	0.002	2.393	1.26–4.52	0.007
VEGF	(−) vs. (+)	1.560	0.62–3.91	0.343	1.356	0.71–2.55	0.347
MVD	(−) vs. (+)	2.218	0.91–5.38	0.078	3.091	0.95–10.1	0.331
Treatment plan	chemotherapy vs. chemoradiotherapy	0.497	0.16–1.49	0.213	0.579	0.28–1.17	0.130

HR, hazard ratio; CI, confidence interval; IPS, international prognostic score; LDH, lactate dehydrogenase; EBER, Epstein-Barr virus-encoded RNA-1 and RNA-2 assessed by *in situ* hybridization; VEGF, vascular endothelial growth factor; MVD, microvessel density.

**Table 4:** Multivariate analysis for overall survival (OS) and event-free survival (EFS).

		OS			EFS		
Covariate	Subcategory	HR	95% CI	*P*-value	HR	95% CI	*P*-value
IPS	<3 vs. ≥3	2.665	1.03–6.83	0.041	1.374	0.74–2.52	0.307
CD68expression	(−) vs. (+)	2.452	0.96–6.25	0.06	2.391	1.27–4.84	0.007
IPS	<3 vs. ≥3	2.160	0.79–5.88	0.132	1.257	0.65–2.42	0.496
CD163expression	(−) vs. (+)	3.009	1.14–7.92	0.026	2.148	1.05–4.36	0.034

HR, hazard ratio; CI, confidence interval; IPS, international prognostic score.

## Discussion

Inflammatory cells such as macrophages, neutrophils, and lymphocytes interact with cancer cells and express angiogenic factors [25], [38], [39]. Specifically, TAMs release a vast variety of proteolytic enzymes, cytokines, inflammatory mediators and growth factors [40]. Of these, members of the VEGF family and angiogenic peptides induce direct angiogenic effects on target endothelial cells or their bone marrow-derived precursors. TAMs also act as ‘bridge cells’ or ‘cellular chaperones’ that guide the fusion of endothelial tip cells for vascular anastomosis and facilitate vascular sprouting [41], [42]. Co-culture with macrophages promote the expressions of VEGF in lung cancer cell lines [38], [43]. In addition, TAMs are closely associated with VEGF expression and MVD in solid tumors [33], [34], [38].

In this study, a significant association of MVD with the expression of CD163 and VEGF was demonstrated in uniformly treated cHL, suggesting that the interaction between host macrophages and HRS cells may synergistically increase angiogenesis in cHL, leading to poor clinical outcome. High CD163 expression was associated with shorter EFS and OS. In contrast, VEGF or MVD did not show significant correlations with survival. Panico et al. also reported the absence of a correlation of MVD with clinical outcomes of cHL [27]. There may be several explanations for this lack of association. Firstly, TAMs may contribute to disease progression through mechanisms other than VEGF secretion or angiogenesis, which may overshadow or negate the effects of angiogenesis. In fact, TAMs contribute to extracellular matrix remodeling, promote cancer cell proliferation, invasion and metastasis; suppress the adaptive immune response [25], [40]. Secondly, VEGF-positive patients are more likely to have the NS or MC disease subtypes, which are associated with a better overall prognosis than other cHL subtypes [44], whereas CD163 expression did not show any such predilection. Thirdly, the relatively small size of the present cohort may preclude the power needed to fully demonstrate the effect of increased TAMs, thereby limiting the interpretation of the present results and calling for further validation.

Our findings confirm the superiority of CD163 as a marker of TAMs. We have shown a correlation between MVD and CD163 expression, but not with CD68 expression, which stands in contrast to the findings by Panico et al [27]. However, Panio et al. used a CD34 antibody for MVD; while we used a CD31 antibody, which is a more sensitive and specific marker of endothelial cell differentiation [45]. In most cancers, TAMs express the M2-like phenotype [25], [46], while CD68 is expressed in both M1 or M2 macrophages [30]. Previous studies yielded conflict results on the effectiveness of CD68 and CD163 expression as a measure of macrophages in cHL tissue. Kamper et al found CD163 to be less effective of CD68 [4]; however Zaki et al found prognostic value in CD163 only [47]. Other studies including the present one found independent prognostic significance for both markers [6], [48]. However, the absence of an association between CD68 expression and MVD in the present study suggests that CD68-positive macrophages have an impact on cHL progression via additional mechanisms other than angiogenesis.

The TAM phenotype is reversible, and these cells can be re-educated to exert antitumor activity [49]–[51]. In a recent study, re-educated CD40-activated macrophages rapidly infiltrated tumors and became tumoricidal in pancreas carcinoma [50]. In addition, angiogenic monocyte subsets can be eliminated by biotherapeutics antibodies as shown in a xenograft model [52], [53]. Anti-VEGF therapy is extensively used in solid malignant tumors [14], [54], [55], and the anti-tumor efficacy of anti-VEGF antibodies has been demonstrated in relapsed HL patients [56]. However, combining chemotherapy with bevacizumab increases the toxicities in patients with diffuse large B-cell lymphoma and peripheral T cell lymphoma [57], [58]. Further studies are warranted to determine the effect of bevacizumab in on cHL patients.

To interpret CD68 and CD163immunostaining, we used a measure of percent positivity as described in most reports [3], [59], rather than an overall visual volume estimation, which is more subject to over- or underestimation due to non-specific staining [48]. It is to be noted, however, that the cutoff values vary among previous studies, albeit in a rather narrow range for most reports. In the pioneering study by Steidl et al., patients were classified into three subgroups based on CD68 expression (<5% positive cells, IHC score 1; 5–25% positive cells, IHC score 2; and >25% positive cells, IHC score 3). While they used 5% as the cutoff in the final analysis, survival was significantly lower in patients with a score of 3 than in those with a score of 2 or 1 [3]. Four other studies also reported inferior survival using a cutoff of 25–30%[59]–[62], which demonstrated the reproducibility of using a 25–30% cutoff. Possible reasons for the inter-study discrepancy include disparate study populations, technical differences, use of tissue microarray vs. whole sections, inter-observer variability, and disparate use of the index of outcome. For example, Steidl et al. used a population enriched with poor-risk patients, whereas we studied a series of consecutive patients in one hospital. While the chemotherapy regimens in most studies were varied, we limited our analysis to patients treated with an ABVD regimen. For survival analysis, while survival was measured in EFS in our previous study [6], here we used OS, as the accuracy of EFS may be limited by innate limitations of radiologic examination. Our use of a more rigorous definition of the limited stage disease, the exclusion of pediatric patients, and the relatively high prevalence of EBV in our study may have contributed to the poorer outcome compared to those of Western studies [63]–[65]. A previous multi-center study in Korea also reported similarly poor survival rates, which may reflect ethnic and socioeconomic differences [66].

Of 32 high-CD68 cases, 20 cases had the high-CD163 expression. In the 32 cases with high-CD68 expression, cases with high-CD163 expression showed inferior OS rate than cases with low-CD163 expression, although there was no statistical significance due to the small sample size (5 year OS rate, 51.3% vs. 91.7%, *P* = 0.183). In multivariate analysis including CD68 and CD163, CD163 index was an independent prognostic marker for or OS (*P* = 0.045), however CD68 index was not an independent prognostic marker for or OS (*P* = 0.582). These results suggest that CD163 positive cells may be a subpopulation of CD68 positive cells although some cases showed more staining CD163 than CD68. CD163 expression particularly was associated with poor prognosis.

Limitations of this study include the retrospective design, short follow-up period, relatively small sample size and TMA-based design of the specimen preparation. TMA design cannot reflect the entire distribution of TAMs because of heterogeneity in TAMs expression with regional variation. Non-specific staining of the inflammatory background by immunophenotypic markers of TAMs also remains a formidable challenge in the clinical quantification of TAMs according to cHL risk stratification. As noted previously [67] background staining was found for both CD68 and CD163, but more so for CD68. Although every effort was made to avoid counting false positives, both CD68 and CD163 counts undoubtedly included minor populations of lymphocytes, basophils, mast cells, and other cell types.

In summary, this study is one of the first to examine the prognostic significance of TAM content in relation to VEGF expression and MVD in a uniformly treated population. Our results show that CD163 expression is associated with poor prognosis and correlates with VEGF expression and MVD, which suggests a role for TAMs in tumor angiogenesis. However, the absence of the prognostic impact of VEGF or MVD suggests that mechanisms other than angiogenesis may also be involved in the contribution of TAMs to tumor progression of cHL. Further studies are warranted to delineate the mechanism of TAM in tumor progression of cHL. Our findings provide evidence supporting new therapeutic approaches, including anti-TAM or anti-VEGF therapy in addition to the current ABVD regimen.

## Supporting Information

Figure S1 **Spearman correlation among CD68, CD163, and MVD.** (A) a positive correlation between CD163 index and MVD (rho = 0.310 and *P*<0.001). (B) No correlation between MVD and indices of CD68.
(TIF)

Figure S2 **Comparison of survival rates according to international prognostic score (IPS).** (A) Overall survival (OS) was significantly worse in the high risk IPS (≥3) group. (B) High risk IPS was not associated with EFS rates.
(TIF)

Table S1 **CD68, CD163 and VEGF index vs. overall survival (OS).**
(DOCX)

Table S2 **Correlations between clinical variables and CD68 expression, CD163 expression, VEGF expression, and MVD.**
(DOCX)
