# Supplementary figures and images for: CD163 Expression Was Associated with Angiogenesis and Shortened Survival in Patients with Uniformly Treated Classical Hodgkin Lymphoma
Source: PLoS One. 2014 Jan 29;9(1):e87066. doi: 10.1371/journal.pone.0087066 (PMC3906082; doi:10.1371/journal.pone.0087066)

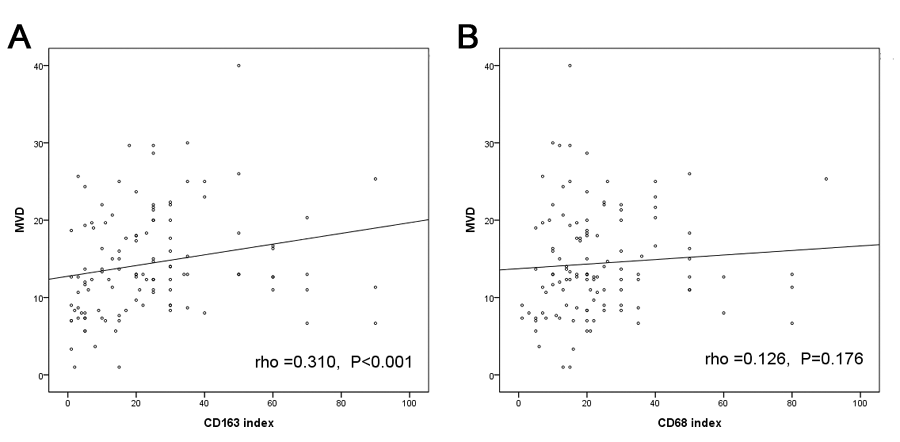

Supplement: Figure S1 — Spearman correlation among CD68, CD163, and MVD. (A) a positive correlation between CD163 index and MVD (rho = 0.310 and P<0.001). (B) No correlation between MVD and indices of CD68. (TIF) [file pone.0087066.s001.tif]

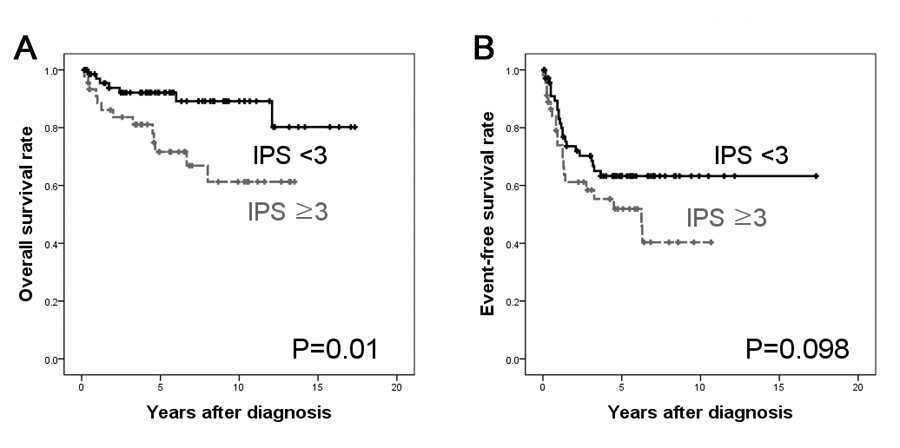

Supplement: Figure S2 — Comparison of survival rates according to international prognostic score (IPS). (A) Overall survival (OS) was significantly worse in the high risk IPS (≥3) group. (B) High risk IPS was not associated with EFS rates. (TIF) [file pone.0087066.s002.tif]
