# Supplementary material for: CD163 Expression Was Associated with Angiogenesis and Shortened Survival in Patients with Uniformly Treated Classical Hodgkin Lymphoma
Source: PLoS One. 2014 Jan 29;9(1):e87066. doi: 10.1371/journal.pone.0087066 (PMC3906082; doi:10.1371/journal.pone.0087066)
Supplement: Table S1 — CD68, CD163 and VEGF index vs. overall survival (OS). (DOCX) [file pone.0087066.s003.docx]

Supplementary Table 1. CD68, CD163 and VEGF index vs. overall survival (OS)

|  | Cutoff (%) | Below cutoff (n) | Above cutoff (n) | *P*-value for OS | Chi^2^-Log-rank for OS |
| --- | --- | --- | --- | --- | --- |
| CD68 | 10 | 16 | 100 | 0.803 | 0.062 |
|  | 15 | 36 | 80 | 0.410 | 0.679 |
|  | 20 | 52 | 64 | 0.079 | 3.007 |
|  | 25 | 76 | 40 | 0.022 | 5.270 |
|  | 30 | 84 | 32 | 0.012 | 6.360 |
|  | 35 | 93 | 23 | 0.013 | 6.169 |
| CD163 | 10 | 30 | 86 | 0.681 | 0.169 |
|  | 15 | 42 | 74 | 0.535 | 0.384 |
|  | 20 | 52 | 64 | 0.114 | 2.498 |
|  | 25 | 65 | 51 | 0.017 | 5.706 |
|  | 30 | 78 | 38 | 0.016 | 5.787 |
|  | 35 | 90 | 26 | <0.001 | 11.51 |
| VEGF | 10 | 79 | 37 | 0.589 | 0.292 |
|  | 15 | 80 | 36 | 0.547 | 0.363 |
|  | 20 | 81 | 35 | 0.440 | 0.597 |
|  | 25 | 83 | 33 | 0.339 | 0.914 |
|  | 30 | 85 | 31 | 0.989 | <0.001 |
|  | 35 | 87 | 29 | 0.812 | 0.056 |
