# Supplementary material for: CD163 Expression Was Associated with Angiogenesis and Shortened Survival in Patients with Uniformly Treated Classical Hodgkin Lymphoma
Source: PLoS One. 2014 Jan 29;9(1):e87066. doi: 10.1371/journal.pone.0087066 (PMC3906082; doi:10.1371/journal.pone.0087066)
Supplement: Table S2 — Correlations between clinical variables and CD68 expression, CD163 expression, VEGF expression, and MVD. (DOCX) [file pone.0087066.s004.docx]

Supplementary table 2. Correlations between clinical variables and CD68 expression, CD163 expression, VEGF expression, and MVD

| Characteristics | CD68 expression | | *P*-value | CD163 expression | | *P*-value | VEGF expression | | *P*-value | Microvessel density | | *P*-value |
| --- | --- | --- | --- | --- | --- | --- | --- | --- | --- | --- | --- | --- |
|  | Low  (<30%)  (n = 84) | High  (≥30%)  (n = 32) |  | Low  (<35%)  (n = 90) | High  (≥35%)  (n = 26) |  | Negative  (≥30%)  (n = 83) | Positive  (≥30%)  (n = 33) |  | Low (<15.33)  (n = 73) | High  (≥15.33)  (n = 43) |  |
| Age |  |  | 0.212† |  |  | <0.001† |  |  | 0.836† |  |  | 0.562† |
| Age <45 | 53 (63.1%) | 16 (50%) |  | 61 (67.8%) | 8 (30.8%) |  | 50 (60.2%) | 19 (57.6%) |  | 45 (61.6%) | 24 (55.8%) |  |
| Age ≥45 | 31 (36.9%) | 16 (50%) |  | 29 (32.2%) | 18 (69.2%) |  | 33 (39.8%) | 14 (42.4%) |  | 28 (38.4%) | 19 (44.2%) |  |
| Gender |  |  | 0.011† |  |  | 0.003† |  |  | 0.302† |  |  | 0.079† |
| Male | 43 (51.2%) | 25 (78.1%) |  | 46 (51.1%) | 22 (84.6%) |  | 46 (55.4%) | 22 (66.7%) |  | 38 (52.1%) | 30 (69.8%) |  |
| Female | 41 (48.8%) | 7 (21.9%) |  | 44 (48.9%) | 4 (15.4%) |  | 37 (44.6%) | 11 (33.3%) |  | 35 (47.9%) | 13 (30.2%) |  |
| Disease subtype |  |  | 0. 448‡ |  |  | 0.342‡ |  |  | 0.077‡ |  |  | 0.319‡ |
| Nodular sclerosis | 59 (70.2%) | 19 (59.4%) |  | 63 (70%) | 15 (57.7%) |  | 50 (60.2%) | 28 (84.8%) |  | 46 (63%) | 32 (74.4%) |  |
| Mixed cellularity | 15 (17.9%) | 7 (21.9%) |  | 16 (17.8%) | 6 (23.1%) |  | 17 (20.5%) | 5 (15.2%) |  | 16 (21.9%) | 6 (14%) |  |
| Lymphocyte-rich | 3 (3.6%) | 2 (6.3%) |  | 4 (4.4%) | 1 (3.8%) |  | 5 (6%) | 0 (0%) |  | 2 (2.7%) | 3 (7%) |  |
| Lymphocyte-  depleted | 1 (1.2%) | 2 (6.3%) |  | 1 (1.1%) | 2 (7.7%) |  | 3 (3.6%) | 0 (0%) |  | 2 (2.7%) | 1 (2.3%) |  |
| Not classifiable | 6 (7.1%) | 2 (6.3%) |  | 6 (6.7%) | 2 (7.7%) |  | 8 (9.6%) | 0 (0%) |  | 7 (9.6%) | 1 (2.3%) |  |
| B symptom |  |  | >0.99† |  |  | 0.476† |  |  | 0.130† |  |  | 0.681† |
| Absent | 57 (67.9%) | 22 (67.2%) |  | 63 (70%) | 16 (61.5%) |  | 53 (63.9%) | 26 (78.8%) |  | 51 (69.9%) | 28 (65.1%) |  |
| Present | 27 (32.1%) | 10 (31.3%) |  | 27 (30%) | 10 (38.5%) |  | 30 (36.1%) | 7 (21.2%) |  | 22 (30.1%) | 15 (34.9%) |  |
| Ann Arbor stage |  |  | 0.833† |  |  | >0.999† |  |  | 0.675† |  |  | 0.694† |
| Limited | 32 (38.1%) | 13 (40.6%) |  | 35 (38.9%) | 10 (38.5%) |  | 31 (37.3%) | 14 (42.4%) |  | 27 (37%) | 18 (41.9%) |  |
| Advanced | 52 (61.9%) | 19 (59.4%) |  | 55 (61.1%) | 16 (61.5%) |  | 52 (62.7%) | 19 (57.6%) |  | 46 (63%) | 25 (58.1%) |  |
| IPS |  |  | 0.02† |  |  | <0.0001 † |  |  | 0.093† |  |  | 0. 115† |
| <3 | 57 (67.9 %) | 14 (43.8%) |  | 63 (70%) | 8 (30.8%) |  | 55 (66.3%) | 16 (48.5%) |  | 49 (67.1%) | 22 (51.2%) |  |
| ≥3 | 27 (32.1%) | 18 (56.3%) |  | 27 (30%) | 18 (69.2%) |  | 28 (33.7%) | 17 (51.5%) |  | 24 (32.9%) | 21 (48.8%) |  |
| LDH (U/L) |  |  | 0.400† |  |  | 0.659 † |  |  | 0.682† |  |  | 0.032† |
| <250 | 33 (39.3%) | 16 (50%) |  | 37 (41.1%) | 12 (46.2%) |  | 34 (41%) | 15 (45.5%) |  | 25 (34.2%) | 24 (55.8%) |  |
| ≥250 | 51 (60.7%) | 16 (50%) |  | 53 (58.9%) | 14 (53.8%) |  | 49 (59%) | 18 (54.5%) |  | 48 (65.8%) | 19 (44.2%) |  |
| EBER |  |  | 0.033† |  |  | 0.005† |  |  | 0.055† |  |  | 0.432† |
| Negative | 58 (69%) | 15 (46.9%) |  | 63 (70%) | 10 (38.5%) |  | 57 (68.7%) | 16 (48.5%) |  | 48 (65.8%) | 25 (58.1%) |  |
| Positive | 26 (31%) | 17 (53.1%) |  | 27 (30%) | 16 (61.5%) |  | 26 (31.3%) | 17 (51.5%) |  | 25 (34.2%) | 18 (41.9%) |  |
| Primary treatment |  |  | 0.107† |  |  | 0.076† |  |  | 0.818† |  |  | 0.665‡ |
| Chemotherapy | 58 (69%) | 27 (84.4%) |  | 62 (68.9%) | 23 (88.5%) |  | 60 (72.3%) | 25 (75.8%) |  | 52 (71.2%) | 33 (76.7%) |  |
| Chemoradiotherapy | 26 (31%) | 5 (15.6%) |  | 28 (31.1%) | 3 (11.5%) |  | 23 (27.7%) | 8 (24.2%) |  | 21 (28.8%) | 10 (23.3%) |  |

VEGF, vascular endothelial growth factor; MVD, microvessel density; IPS, international prognostic score; LDH, lactate dehydrogenase; EBER, Epstein-Barr virus-encoded RNA-1 and RNA-2 assessed by *in situ* hybridization

†Chi-squared test by two-sided Pearson’s test.

‡ Chi-squared test by two-sided Fisher’s test.
